# The Beneficial Effects of Cognitive Walking Program on Improving Cognitive Function and Physical Fitness in Older Adults

**DOI:** 10.3390/healthcare9040419

**Published:** 2021-04-05

**Authors:** Suh-Jung Kang, Byung-Hoon Kim, Hyo Lee, Jinsung Wang

**Affiliations:** 1 Sports and Health Care Major, College of Culture and Arts, Sangmyung University, Seoul 03016, Korea; hyolee@smu.ac.kr; 2 Sports Science Research Center, Sangmyung University, Seoul 03016, Korea; kimbang79@hanmail.net; 3 Department of Kinesiology, University of Wisconsin-Milwaukee, Milwaukee, WI 53211, USA; wang34@uwm.edu

**Keywords:** walking, cognitive function, cardiorespiratory fitness, elderly health

## Abstract

Exercise and cognitive training can improve the brain-related health of the elderly. We investigated the effects of a cognitive walking program (CWP) involving simultaneous performance of indoor walking and cognitive training on cognitive function and physical fitness compared to normal walking (NW) outdoors. Participants were grouped according to whether they performed regular exercise for at least 3 months prior to the participation in this study. Active participants were assigned to the CWP-active group (CWPAG). Sedentary participants were randomly assigned to the CWP (CWPSG) or NW group (NWSG). CWP and NW were performed for 60 min, 3 times a week, for 6 months. Cognitive function (attention, visuospatial function, memory, and frontal/executive function) and physical fitness (cardiorespiratory fitness, lower extremity muscular strength, and active balance ability) were measured at baseline, 3 months, and 6 months after the program onset. Cognitive function showed improvements over time in all three groups, especially in CWPAG. No clear difference was observed between CWPSG and NWSG. Improvements in all fitness measures were also observed in all three groups. These findings collectively indicate the beneficial effects of CWP, as well as NW, on improving cognitive function and physical fitness in older adults, especially those who are physically active.

## 1. Introduction

Previous studies have demonstrated that physical activity can improve the function of various body systems, including cardiovascular, metabolic, endocrine, and skeletal systems [1,2]. In addition, physical activity has been repeatedly shown to prevent neurodegenerative diseases such as Alzheimer’s disease and improve cognitive function [3,4].

Walking is a complex process involving the interaction of neuromuscular, sensory, and cognitive functions [5], and walking ability is related to improved cognitive and executive function [6]. Many studies have shown that participation in walking exercises may help to prevent cognitive decline and lower the risk of dementia. For example, regular long-term walking in elderly women improved cognitive function and lowered the level of cognitive decline [7]. In addition, older adults who participated in brisk walking for 2 years showed relatively improved cognitive function compared to those who did not participate [8], and increased walking was associated with a lower incidence and risk of dementia [9,10]. However, contradictory findings have also been reported. For example, Eggermount et al. showed that walking exercise had no beneficial effects for improving cognitive function in individuals with cognitive impairment [11], and the same authors also reported that walking has minimal or no effect when cardiovascular risk factors are involved [12].

Cardiorespiratory fitness (CRF), which refers to the ability of the circulatory and respiratory systems to supply oxygen to skeletal muscles during sustained physical activity, generally decreases with age [13]. However, elderly populations with better CRF were able to perform better in cognitive tasks [14], especially in tasks requiring executive functions [15]. Evaluation of executive function requires simultaneous performance of dual-tasks and two additional tasks, and a dual-task paradigm has been suggested as a useful method to assess the relationship between brain function and CRF [16]. Based on the findings of previous studies, exercise, cognitive function, and physical fitness or dual-task performance level are related, and it is thought that a program that combines exercise and cognitive training would have beneficial effects on cognitive function in the elderly. In fact, sequential [17] and simultaneous [18] performance of dual-task cognitive training improved cognitive function, although a meta-analysis indicated that the overall effect sizes for combined interventions versus control groups or physical practice alone were only 0.29 and 0.22, respectively, which are relatively small effects [19].

These findings indicate a need to further develop combined training methods that are more effective than physical practice alone. Therefore, we developed a cognitive walking exercise program (CWP), which is a walking-based indoor exercise program designed to improve cognitive function and fitness in the elderly. CWP is based on the idea of square stepping exercise [20] but also includes other exercise and cognitive training such as dual-tasks and visual memory. The dual-task program in CWP requires physical movements of the upper and lower body, rather than language or arithmetic used in most cognitive training, which may lead to additional benefits due to its game-like features.

Therefore, the purpose of this study was to assess the effects of CWP on cognitive function and physical fitness in the elderly compared to normal walking (NW). The specific aims of this study were as follows: (1) to assess the effects of CWP and NW on cognitive function and physical fitness, (2) to assess the effects of CWP on active and sedentary participants, and (3) to assess the time required for changes in cognitive function and physical fitness to occur.

## 2. Methods

### 2.1. Participants

Participants were recruited through two community health care centers in Seoul, Korea. The inclusion criteria were as follows: (1) at least 65 years old and (2) normal cognitive function as assessed through Korean mini-mental state examination (K-MMSE). The exclusion criteria were as follows: (1) a recent history of severe cardiovascular disease, (2) neurological disease or peripheral disorder affecting movement of their arms and legs, (3) significant orthopedic conditions limiting mobility, (4) visual impairment, (5) probable dementia as assessed by K-MMSE (score < 24), and (6) any other factors that could potentially limit the ability to fully participate in the intervention (e.g., musculoskeletal problems, severe depression, plans to leave out of town for more than 4 days in a row during the participation period, etc.).

After recruitment, participants were classified as active or sedentary according to the Stanford Brief Active Survey (SBAS) [21]. Active participants were assigned to the CWP-active group (CWPAG), and sedentary participants were randomly assigned to the CWP-sedentary group (CWPSG) or normal walking sedentary group (NWSG). At baseline (T0) and after 3 months (T1), there were a total of 75 participants (CWPAG = 31, CWPSG = 29, NWSG = 15, N = 75). After 6 months (T2), 23 participants had dropped out of the study for personal reasons (surgery, family problems, injuries, etc.) Therefore, data from 52 participants (CWPAG = 20, CWPSG = 21, NWSG = 11, N = 52) were used for the final analysis (Table 1). Table 1 also shows the physical characteristics of the participants. We measured the body mass index and waist circumference to see if the subject groups had any differences in terms of their physiques.

All participants signed the consent form prior to the participation in this study, which was approved by the Institutional Review Board of Sangmyung University.

### 2.2. Exercise Programs

The subjects who were assigned to CWP performed the physical activity in indoor classrooms at two community health care centers in Seoul, and those assigned to NW performed the activity on outdoor trails near the two centers between 2018 and 2019. A total of six exercise classes were offered at the two health care centers for 2 years. Each exercise program was conducted three times a week for 60 min per session for 6 months.

#### 2.2.1. Cognitive Walking Program

**Figure 1:**
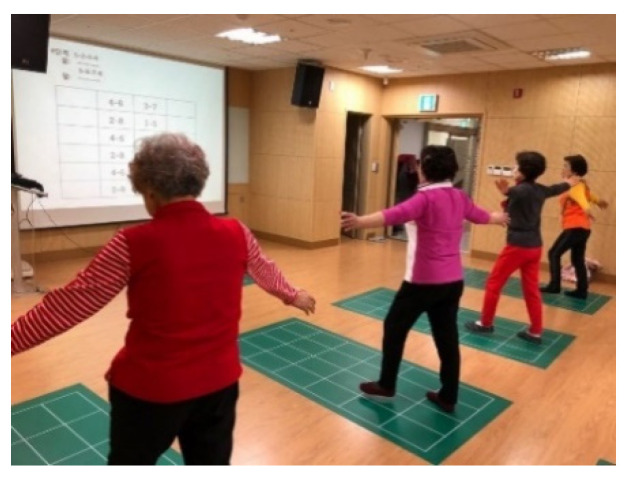
Visual memory program in cognitive walking program (CWP).

CWP includes both walking exercise and cognitive training programs and is performed indoors using a specially designed rubber mat. The mat consists of 24 cells (6 × 4 cells, 180 × 80 cm) with a number assigned to each cell, and the participants are asked to walk forward, backward, and diagonally according to the numbers shown on the board placed at the front of the room (Figure 1), which indicate which foot should be placed in which cell in a proper sequence [22].

CWP consists of three types of walking: cardiorespiratory walking, dual-task walking, and visual memory walking. Cardiorespiratory walking involves walking patterns that are focused on improving CRF by moving the lower limbs rigorously and widely (e.g., squat, lunge, and ad/abduction). Additionally, bending and straightening the knees to strengthen the lower extremity muscles and side-step motions to change the center of gravity are included. Dual-task walking involves walking patterns that require the upper and lower limbs to perform specific movements concurrently. (Here, a dual task is defined as a task that requires coordination, maintenance, and integration of two tasks [16,23]). Visual memory walking involves presenting various levels and stages of walking patterns to the participants and having them perform the exercise based on memory (Figure 1). Three levels of stepping patterns are available for each type of walking: beginner, intermediate, and advanced, and each level consists of 15 stages, thus providing 45 different stepping patterns. The difficulty of the 45 stepping patterns varies based on the complexity of the arm and/or leg movements required to perform within each stepping pattern. All participants in CWP started with the easiest stepping pattern (beginner, stage 1) for each type of walking in the very first session, and they advanced to the next stage/level for each walking type once they mastered the given stepping pattern. The subjects were instructed to maintain a moderate intensity (Borg rating of perceived exertion (RPE scale: 12–13).

To determine the feasibility of this program, we had conducted a pilot study, in which we had 10 older adults participate in CWP for 2 weeks (60 min/session, 3 sessions/week). Following the 2-week program, all participants filled out a survey and had an interview with the experimenter. The survey consisted of 10 questions, which asked whether the intensity of CWP seemed appropriate, whether it seemed to have beneficial effects for improving balance, muscular strength, and cardiorespiratory fitness and also for improving memory and cognitive function, whether it was fun enough to continue participating, etc., and the responses were assessed using a 5 Likert scale. The overall responses were very positive, based on which we decided to conduct our current study.

#### 2.2.2. Normal Walking Program

The effects of CWP were compared to those of NW, which was performed on outdoor trails. Participants assigned to the NWSG walked together with an instructor. Walking speed was adjusted by the instructor to maintain moderate to somewhat-hard exercise intensity for the participants (RPE scale: 12–13).

### 2.3. Measurements

#### 2.3.1. Physical Characteristics

Body mass index (BMI) was determined using body mass and height measurements, and waist circumference was measured.

#### 2.3.2. Cognitive Function

Cognitive function was evaluated using the Seoul Neuropsychological Screening Battery (SNSB-II) [24,25] by certified clinical psychologists who had at least 3 years of experience with administering the SNSB-II. Scores for attention, visuospatial function, memory, and frontal/executive function were calculated, and T-scores of each variable were used to analyze these factors.

#### 2.3.3. Fitness Test

CRF, lower extremity muscle strength, and active balance ability were measured to evaluate the physical fitness of the participants [26]. Cardiorespiratory fitness was evaluated by measuring the number of times the knee was lifted by 70° for 2 min. Lower extremity muscle strength was measured by the number of times the participants sat and stood up completely from a chair for over 30 s. Active balance ability was measured by the time required to run to a target located 3 m ahead, starting from a seated position and then returning to a seated position on the chair.

#### 2.3.4. Measurement Frequency

All variables were measured at baseline (T0) and at 3 (T1) and 6 months (T2) thereafter.

### 2.4. Statistical Analysis

A series of one-way ANOVAs were performed to see if there was any significant difference among the three subject groups in terms of their physical characteristics (age, BMI, waist circumferences). To test the intervention effects, a mixed-factors ANOVA was performed with group as a between-subjects factor and time as a within-subjects factor. If the interaction effect was found to be statistically significant, contrasts of marginal linear predictions were performed as post hoc analyses to test changes in group differences over time. For cognitive function, changes in outcome variables over time within each subject group were examined using contrasts of marginal linear predictions as a priori analyses (i.e., regardless of whether the group main or interaction effects were significant or not).

## 3. Results

One-way ANOVAs indicated that the group main effect was not significant for any of the physical characteristics (age, BMI, waist circumferences).

### 3.1. Cognitive Function

Table 2 shows the detailed results of the ANOVA for the four factors of cognitive function in each subject group measured at T0, T1, and T2. Neither the main effect of group nor the group × time interaction was significant for any of the four factors. The main effect of time was significant for all factors except attention, indicating improvements over time in general.

A priori comparisons revealed significant differences over time within each group. As shown in Table 2, attention was significantly different between T0 and T1 and between T0 and T2 in CWPAG. Visuospatial function was significantly different between T0 and T2 in both CWPAG and CWPSG. Memory was significantly different between T0 and T1 and between T0 and T2 in all three groups. Frontal/executive function was significantly different between T0 and T2 in a marginal manner in CWPAG, and between T0 and T2 in NWSG.

### 3.2. Fitness Tests

The ANOVA results for three measures of physical fitness in each subject group assessed at T0, T1, and T2 are provided in Table 3. Significant group × time interactions were found for CRF and active balance ability. Post hoc analyses indicated a significant difference between T0 and T1, and between T0 and T2 for all three groups, but at different rates, in terms of both CRF and active balance ability. For lower extremity muscle strength, the main effect of time was significant, indicating an improvement over time. Neither the main effect of group nor the group × time interaction was significant for lower extremity muscle strength.

## 4. Discussion

In the current study, we aimed to assess the effects of CWP, compared to NW, on cognitive function and physical fitness in older adults. The main finding of this study is that regular performance of CWP can cause long-term improvements in the cognitive function and physical fitness of the elderly. More factors of cognitive function were improved at T2 than at T1, and additional improvement was observed at T2. Similar effects were observed in the NWSG.

CWP was originally developed based on the square stepping exercise (SSE) [20]. In a previous study, SSE improved physical strength and cognitive function in the elderly [27]. However, CWP differs from SSE in that it includes dual-task programs that simultaneously use lower body and upper body motions to perform stepping for cognitive training. In addition, the program includes visual memory training, which involves memorizing the contents of the program at various difficulty levels and recalling the sequence of memorized stepping. Including cognitive function training benefited CWP participants by improving cognitive function and feelings of challenge and achievement during the course of the exercise program [22].

In our study, the group × time interaction for cognitive function was not significant. Because of that, we cannot determine whether CWP is more beneficial than NW in terms of its effect on improving cognitive function. However, in CWPAG, all four factors of cognitive function improved over time, and in CWPSG, visuospatial function and memory improved. These findings suggest that dual-task training and visual memory training incorporated into CWP play a positive role in improving cognitive function.

Dual tasks involve attention and executive function processes. It is thought that the elderly have deteriorated central processing function [28], which is why their dual-task performance ability is lower than that of young adults [29]. Moreover, analysis of age, mobility, and cognitive performance has shown that age negatively affected mobility and cognitive performance. It has also been suggested that dual-task training would be useful to improve cognitive and motor skills [30], and simultaneous performance of exercise and cognitive training were more effective than cognitive training or exercise alone [31]. Our findings are partly in line with this argument by demonstrating that the simultaneous performance of exercise and cognitive training incorporated in CWP improved the cognitive function of older adults tested in this study.

With respect to the time required for changes in cognitive function, it varied across the subject groups and also across the four factors of cognitive function. Improvements in attention and visuospatial function were observed at T1 and T2, respectively, in CWPAG; those in visuospatial function were observed at T2 in CWPSG. Improvements in memory were observed at T1 in all three groups. Finally, it took 6 months (T2) to observe an improvement in frontal/executive function in CWPAG and NWSG. These findings suggest that CWP can demonstrate its beneficial effects for improving certain features of cognitive function as early as 3 months following the participation in regular exercise. It is interesting to note here that cognitive function was improved in both CWPSG and CWPAG. In fact, it is CWPAG who benefited the most from participating in our exercise program in terms of improving cognitive function, in that this is the only group who showed improvement in all four factors of cognitive function. Given that the participants in CWPAG have already been exercising prior to their participation in this study, this finding suggests that while CWP is beneficial for all, its beneficial effect may be greater for those who exercise regularly for a longer period of time.

In our study, we did not analyze the mechanism underlying cognitive function improvement. However, previous studies have explained that participation in exercise can improve cognitive function through neurophysiological changes, and these findings support the results of our study. General aging leads to decreased volume of the corpus callosum [32], functional changes [33], and decreased volume [34] of the hippocampus, which is a brain region related to learning and decreased neuronal regeneration and plasticity [35]. As a result, these changes in brain structure and function decrease cognitive function [36]. However, regular exercise over time increases the production of brain-derived neurotrophic factor and nerve growth factor, which leads to changes in neuronal cells and increases neurogenesis [37,38]. These previous studies have shown that long-term exercise can induce positive changes in the brain, and the results of our study may be attributed to such changes. In particular, performing exercise and cognitive training at the same time improved neurogenesis and angiogenesis and upregulated neurotrophic factor [39], and cognitive training increased neurons and neuronal networks [40]. Thus, exercise and cognitive training incorporated together in CWP might have shown synergistic effects [41] to improve cognitive function.

With regard to the changes in physical fitness, improvements in CRF and active balance ability were observed at T1, and those in lower extremity muscular strength at T2 in all three groups. These findings indicate that at least 3 months may be required to improve certain features of physical fitness and that both CWP and NW are effective exercise methods for improving physical fitness. In particular, improved physical fitness in our study may be related to improved cognitive function. Lower extremity muscular strength [42] and grip strength [43,44] are related to cognitive ability. Lower extremity muscular strength is a key physical factor that can affect mobility, which is also related to cognitive ability [45]. In addition, higher CRF is related to higher brain function [46], less structural brain atrophy [47], greater brain activation, and better cognitive function [13,48]. A meta-analysis study reported that exercise intervention is effective in improving cognitive function [49]. Based on these previous findings, it is thought that improved physical fitness following CWP and NW had positive effects on the improvement in cognitive function.

In this study, the effect size (partial η^2^) for significant main effects of time (*p* < 0.05) for visuospatial function, memory, and frontal/executive function were 0.08, 0.481, and 0.113, respectively (Table 2), which are medium to large effect sizes [50]. The effect size for significant main effects of time for cardiorespiratory fitness, leg muscular strength, and balance were 0.434, 0.173, and 0.599, respectively, which are large effect sizes. These results indicate that the intensity and duration of the exercise programs employed in the current study are adequate to improve both cognitive function and physical fitness in older adults regardless of the type of the program (i.e., CWP, NW).

This study has several limitations. First, we did not include any control group whose members participated in some programs other than physical activity. Thus, while it is reasonable to assume that the significant changes observed across different time points within each subject group can be attributed to CWP or NW, it is possible (though unlikely) that those changes may be attributed to certain factors other than the exercise programs per se (e.g., passage of time). Second, we were not able to detect a significant difference between subject groups, probably due to lack of statistical power. It would have been ideal if we tested additional subjects, but we were not able to do so because of the pandemic caused by COVID-19. Third, both CWP and NW were performed for 60 min, 3 times a week, for 6 months. However, the two programs may have some differences other than the involvement of cognitive demands (e.g., the number of steps), which may have contributed to the observed changes in each group differently. In addition, we did not assess the physical and physiological demands of each level of exercise in each subject group. The use of heart rate monitors or triaxial accelerometers during the activity of each program would have provided more objective assessments of the physical and physiological demands of each exercise program. Finally, SNSB-II, which was used for cognitive testing, was administered repeatedly, and its learning effect may have affected the outcome. Moreover, we did not test for intra- or inter-observer reliability for administering SNSB-II in this study, although we tried to minimize inter-observer variability by having the same observer test the same subjects across all three assessments of the SNSB-II. We also tried to have the same observer test as many subjects as possible.

## 5. Conclusions

In the present study, significant changes in terms of cognitive function and physical fitness were observed across three different time points following the subjects’ participation in both CWP, which involves indoor walking and cognitive training, and NW, which involves typical walking outdoors. We did not observe a significant difference between the two exercise programs, possibly due to lack of statistical power. However, our findings confirm that CWP is an effective exercise method that can improve both cognitive function and physical fitness, especially for older adults who exercise regularly. These findings suggest that an exercise program similar to the CWP employed in the current study, which requires performing cognitively stimulating patterns of upper and lower limb movements together, can be used for older adults who prefer performing physical activities indoors. Future studies are warranted to determine the beneficial effects of CWP in individuals of varying ages (e.g., middle-aged vs. older adults), especially those with mild cognitive impairment.

## Figures and Tables

**Table 1:** Characteristics of study participants.

Group	Age (years)	Body Mass Index (kg/m^2^)	Waist Circumference (cm)
CWPAG (n = 20)	72.40 ± 3.90	24.2 ± 1.64	86.59 ± 5.19
CWPSG (n = 21)	73.29 ± 4.80	24.42 ± 2.34	86.34 ± 8.41
NWSG (n = 11)	74.45 ± 4.47	25.53 ± 2.13	89.95 ± 6.42

Results are expressed as mean ± standard deviation. CWPAG: cognitive walking program-active group; CWPSG: cognitive walking program-sedentary group; NWG: normal walking-sedentary group.

**Table 2:** Comparison of cognitive function.

Cognitive Function	Group	T0	T1	T2	Group	Time	Group × Time Interaction
Attention	CWPAG	49.97 ± 9.17	53.33 ± 8.291 ^1^	54.15 ± 8.35 *	F(2) = 0.68, *p* = 0.510, partial η^2^ = 0.066	F(2) = 1.3, *p* = 0.277, partial η^2^ = 0.025	F(4) = 0.91, *p* = 0.461, partial η^2^ = 0.034
	CWPSG	48.71 ± 8.22	50.06 ± 11.28	50.93 ± 9.87			
	NWSG	52.17 ± 8.47	51.93 ± 8.9	51.71 ± 7.24			
Visuospatial function	CWPAG	39.58 ± 17.64	45.12 ± 15.73	50.87 ± 18.8 **	F(2) = 2.41, *p* = 0.097 partial η^2^ = 0.013	F(2) = 4.45, *p* = 0.014, partial η^2^ = 0.08	F(4) = 1.53, *p* = 0.198, partial η^2^ = 0.056
	CWPSG	46.32 ± 15.08	52.87 ± 7.7	53.99 ± 12.8 *			
	NWSG	49.97 ± 11.16	55.52 ± 6.28	48.48 ± 15.66			
Memory	CWPAG	52.95 ± 8.04	55.85 ± 9.35 *	61.18 ± 12.12 ***	F(2) = 0.38, *p* = 0.682, partial η^2^ = 0.046	F(2) = 47.77, *p* < 0.001, partial η^2^ = 0.481	F(4) = 0.91, *p* = 0.464, partial η^2^ = 0.034
	CWPSG	48.89 ± 9.83	56.37 ± 11.42 **	60.33 ± 10.21 ***			
	NWSG	46.68 ± 6.14	55.74 ± 6.07 ***	58.43 ± 6.34 ***			
Frontal/ executive function	CWPAG	56.26 ± 12.52	57.37 ± 12.77	59.45 ± 14.42 ^2^	F(2) = 0.15, *p* = 0.863, partial η^2^ = 0.035	F(2) = 6.55, *p* = 0.002, partial η^2^ = 0.113	F(4) = 0.97, *p* = 0.425, partial η^2^ = 0.036
	CWPSG	57.88 ± 10.11	59.48 ± 11.27	60.02 ± 11.77			
	NWSG	51.7 ± 16.66	52.75 ± 16.48	58.3 ± 17.59 **			

Results are expressed as mean ± standard deviation. CWPAG: cognitive walking program-active group; CWPSG: cognitive walking program-sedentary group; NWG: normal walking-sedentary group. Significance level of the contrasts of marginal linear predictions vs. Time 0: ^1^ *p* = 0.051, ^2^ *p* = 0.057, * *p* < 0.05, ** *p* < 0.01, *** *p* < 0.001.

**Table 3:** Comparison of physical fitness.

Physical Fitness Components	Group	T0	T1	T2	Group	Time	Group × Time Interaction
Cardiorespiratory fitness	CWPAG	113.9 ± 16.41	120.7 ± 10.47 *	135.65 ± 10.52 ***	F(2) = 0.52, *p* = 0.599, partial η^2^ = 0.025	F(2) = 39.82, *p* < 0.001, partial η^2^ = 0.434	F(4) = 4.26, *p* = 0.003, partial η^2^ = 0.141
	CWPSG	111.71 ± 19.4	124.05 ± 15.15 **	125.48 ± 22.46 **			
	NWSG	97.27 ± 10.94	126.82 ± 6.87 ***	127 ± 13.04 ***			
Lower extremity muscular strength	CWPAG	16.65 ± 2.62	19.6 ± 3.89	24.8 ± 5.05 **	F(2) = 0.24, *p* = 0.79, partial η^2^ = 0.004	F(2) = 10.91, *p* < 0.001, partial η^2^ = 0.173	F(4) = 0.39, *p* = 0.817, partial η^2^ = 0.015
	CWPSG	17.67 ± 3.38	19.9 ± 3.02	27.24 ± 26.35 **			
	NWSG	13.45 ± 2.66	20.55 ± 2.7	22.55 ± 2.5 *			
Active balance ability	CWPAG	7.03 ± 0.98	6.56 ± 1.02 **	5.56 ± 0.56 ***	F(2) = 1.35, *p* = 0.266, partial η^2^ = 0.069	F(2) = 77.65, *p* < 0.001, partial η^2^ = 0.599	F(4) = 4.04, *p* = 0.004, partial η^2^ = 0.134
	CWPSG	7.37 ± 1.16	6.5 ± 0.78 **	5.8 ± 0.74 ***			
	NWSG	7.82 ± 0.9	5.88 ± 0.69 ***	5.73 ± 0.74 ***			

Results are expressed as mean ± standard deviation. CWPAG: cognitive walking program-active group; CWPSG: cognitive walking program-sedentary group; NWG: normal walking-sedentary group. Significance level of the contrasts of marginal linear predictions vs. Time 0: * *p* < 0.05, ** *p* < 0.01, *** *p* < 0.001.

## Data Availability

Data are freely available at https://osf.io/gqmw5 (accessed on 8 March 2021).
